# Polypharmacy Reflects Metabolic Burden Rather than Frailty in Older Adults with Type 2 Diabetes: A Comprehensive Geriatric Assessment Study

**DOI:** 10.3390/jcm15124674

**Published:** 2026-06-16

**Authors:** Funda Datlı Yakaryılmaz, Ayten Eraydın

**Affiliations:** 1 Department of Internal Medicine, Division of Geriatrics, Faculty of Medicine, İnönü University, Malatya 44280, Türkiye; 2 Department of Internal Medicine, Division of Endocrinology and Metabolism, Faculty of Medicine, Pamukkale University, Denizli 20070, Türkiye; aeraydin@pau.edu.tr

**Keywords:** polypharmacy, type 2 diabetes mellitus, frailty, comprehensive geriatric assessment, HbA1c

## Abstract

**Background:** Polypharmacy is highly prevalent among older adults with type 2 diabetes mellitus (T2DM) and is traditionally considered a marker of geriatric vulnerability. However, it remains unclear whether polypharmacy is more closely associated with multidimensional frailty or metabolic burden in this population. **Methods:** In this retrospective cross-sectional study, 278 adults aged ≥65 years with T2DM underwent comprehensive geriatric assessment (CGA), including evaluation of functional status, cognition, nutrition, depressive symptoms, frailty, and physical performance. Frailty was assessed using the Fried phenotype. Polypharmacy was defined as the concurrent use of ≥5 medications. Multivariable logistic regression and interaction analyses were performed to identify independent predictors of polypharmacy. Receiver operating characteristic (ROC) analyses were conducted to evaluate the discriminative performance of metabolic parameters. **Results:** Polypharmacy was present in 54.7% of participants. Patients with polypharmacy had significantly higher HbA1c and fasting glucose levels compared with those without polypharmacy (both *p* < 0.001). In multivariable analysis, higher HbA1c levels remained independently associated with polypharmacy (OR = 4.99, 95% CI: 3.18–7.84, *p* < 0.001), whereas frailty status was not significantly associated with polypharmacy (OR = 0.58, 95% CI: 0.15–2.21, *p* = 0.427). No significant interaction was observed between HbA1c and frailty status (*p* for interaction > 0.05). Among CGA domains, only functional status and gait speed differed in unadjusted analyses, while cognition, nutritional status, and depressive symptoms were not significantly associated with polypharmacy after adjustment. HbA1c demonstrated strong discriminative performance for polypharmacy (AUC = 0.898, 95% CI: 0.863–0.931), with an optimal cut-off of 6.81%. **Conclusions:** In older adults with T2DM, polypharmacy appeared to be more closely associated with markers of poor glycemic control, particularly HbA1c levels, than with frailty status itself. These findings suggest that medication burden in older adults with T2DM may reflect treatment intensification and suboptimal glycemic control in addition to geriatric vulnerability.

## 1. Introduction

Polypharmacy has become a central issue in the care of older adults, largely driven by increasing life expectancy, multimorbidity, and the widespread implementation of disease-specific clinical guidelines. The concurrent use of multiple medications is highly prevalent in geriatric populations and has been consistently associated with adverse drug reactions, functional decline, hospitalization, and mortality [1,2]. Although polypharmacy is commonly defined as the use of five or more medications, it is increasingly recognized as a multidimensional construct that may reflect not only medication burden but also clinical complexity and vulnerability [3].

In older adults with type 2 diabetes mellitus (T2DM), polypharmacy is particularly pronounced due to the coexistence of cardiometabolic comorbidities and the progressive intensification of glucose-lowering therapies. Current guidelines emphasize individualized glycemic targets in older populations; however, inadequate glycemic control frequently leads to treatment escalation and increased medication exposure over time [4,5]. Consequently, polypharmacy in this population may represent both therapeutic complexity and disease severity rather than a single underlying mechanism. At the same time, aging is associated with a progressive decline in physiological reserve, manifesting as impairments in muscle strength, mobility, cognition, and nutritional status. Objective measures such as handgrip strength, gait speed, and calf circumference are widely used indicators of sarcopenia and physical performance, while comprehensive geriatric assessment (CGA) domains including functional status, cognitive function, and nutritional status provide a multidimensional evaluation of vulnerability in older adults [6,7,8]. These parameters are strongly associated with adverse clinical outcomes and are increasingly incorporated into risk stratification in geriatric medicine.

Frailty is a clinically relevant geriatric syndrome characterized by decreased physiological reserve and increased vulnerability to stressors, and is strongly associated with adverse outcomes including falls, hospitalization, and mortality. The Fried frailty phenotype, which operationalizes frailty through five components, unintentional weight loss, exhaustion, low physical activity, slow gait speed, and weak handgrip strength, remains the most widely validated frailty assessment tool in geriatric research. Individuals meeting one or two criteria are classified as pre-frail, while those meeting three or more are considered frail [9]. Previous studies have shown that polypharmacy may cluster with frailty, disability, and other geriatric syndromes; however, the direction and underlying drivers of this relationship remain unclear [2,3,10]. In older adults with T2DM, the coexistence of frailty and polypharmacy may represent both a consequence of disease-related treatment intensification and an independent marker of vulnerability [11]. In particular, it is uncertain whether polypharmacy in this population primarily reflects geriatric vulnerability characterized by reduced physiological reserve, or whether it is more closely related to metabolic dysregulation and treatment intensification. Clarifying this distinction is clinically important, as it may influence therapeutic decision-making, including medication optimization and deprescribing strategies in older adults.

Therefore, the aim of this study was to evaluate the relationship between polypharmacy and multidimensional domains of comprehensive geriatric assessment—including frailty status assessed by the Fried phenotype—in older adults with T2DM. In addition to standard CGA components, we incorporated objective indicators of physical reserve—including handgrip strength, gait speed, calf circumference, and balance gait performance—to better characterize the clinical profile associated with medication burden. We hypothesized that polypharmacy would be more strongly associated with metabolic burden than with frailty status alone.

## 2. Materials and Methods

### 2.1. Study Design and Setting

This retrospective cross-sectional study was conducted at the Internal Medicine and Geriatrics outpatient clinics of İnönü University Turgut Özal Medical Center, a tertiary referral hospital. The study was designed and reported in accordance with the Strengthening the Reporting of Observational Studies in Epidemiology (STROBE) guidelines.

### 2.2. Study Population

Patients aged ≥65 years with a diagnosis of type 2 diabetes mellitus (T2DM) who were evaluated between February 2021 and November 2021 were screened for eligibility.

**Inclusion criteria:**

- Age ≥ 65 years,
- Diagnosis of T2DM,
- Availability of complete comprehensive geriatric assessment (CGA) data,
- Availability of laboratory measurements including HbA1c.

**Exclusion criteria:**

- Missing key laboratory or CGA data,
- Acute illness at the time of evaluation,
- Known active malignancy or terminal illness,
- Acute delirium, severe communication impairment, or inability to complete comprehensive geriatric assessment procedures.

After applying eligibility criteria, 299 patients were initially included. However, 278 patients with complete datasets available for all study variables were included in the final analysis.

### 2.3. Data Collection

Demographic characteristics, comorbidities, laboratory parameters, medication use, and CGA data were extracted from the electronic hospital information system.

### 2.4. Definitions

Polypharmacy was defined as the concurrent use of ≥5 medications, consistent with widely accepted definitions in geriatric research. Hyperpolypharmacy was defined as the use of ≥10 medications and was evaluated in sensitivity analyses. Medication count included prescribed medications recorded in the hospital information system. Over-the-counter drugs and dietary supplements were not systematically available in the retrospective records and therefore were not included in the formal medication count.

Poor glycemic control was defined as HbA1c > 7.5%. This threshold was selected as a standardized analytical cut-off to characterize metabolic burden across the cohort and was based on ADA recommendations suggesting HbA1c targets around 7.0–7.5% for healthy or functionally preserved older adults with diabetes [4]. However, the threshold was not intended to represent an individualized therapeutic target for all participants. Current guidelines recommend tailoring glycemic targets according to frailty status, functional capacity, cognitive function, comorbidity burden, and life expectancy [4]. Because of the retrospective design of the study, individualized HbA1c targets and clinician-specific treatment goals were not consistently available. Therefore, a uniform HbA1c threshold was applied to allow comparability between frail and non-frail participants and to evaluate the relationship between metabolic burden and polypharmacy.

Frailty was assessed using the Fried frailty phenotype, which includes five components: unintentional weight loss, self-reported exhaustion, low physical activity, slow gait speed, and weak handgrip strength [9]. Low physical activity was determined based on reduced self-reported habitual daily activity documented during routine geriatric assessment. Handgrip strength was measured using a digital Takei hand dynamometer and recorded in kilograms. Measurements were performed with the participant in a seated position and the elbow flexed at approximately 90°. Three consecutive measurements were obtained, and the highest value was used for analysis. Although frailty classification was based on the Fried phenotype, low muscle strength was interpreted according to EWGSOP2 recommendations as <16 kg for women and <27 kg for men [6]. Gait speed was assessed using a 4 m walking test performed at usual pace and expressed in meters per second [7]. Calf circumference was measured at the widest part of the calf, and values < 31 cm were considered indicative of low muscle mass according to EWGSOP2 recommendations [6]. Calf circumference was not used as a diagnostic component of frailty classification but was evaluated as an additional marker of muscle reserve and physical vulnerability.

### 2.5. Comprehensive Geriatric Assessment

All patients underwent a comprehensive geriatric assessment (CGA) including evaluation of functional status, cognitive performance, depressive symptoms, and nutritional status.

**Functional status**: Functional independence in activities of daily living was assessed using the Barthel **Activities of Daily Living (ADL)** Index, which evaluates functional performance in basic daily activities and generates a score ranging from 0 to 100, with higher scores indicating greater independence [12].

Instrumental activities of daily living were assessed using the Lawton Instrumental Activities of Daily Living (IADL) scale, which evaluates abilities such as telephone use, shopping, transportation, medication management, and financial management [12].

**Cognitive function**: Cognitive status was evaluated using the Standardized Mini-Mental State Examination (SMMSE) [13]. Patients with cognitive impairment were not systematically excluded, as cognitive dysfunction represents an important component of geriatric vulnerability in older adults with T2DM. However, individuals unable to participate reliably in comprehensive geriatric assessment because of acute delirium or severe communication impairment were excluded from the study.

**Depressive symptoms**: Depressive symptoms were evaluated using the **15-item Geriatric Depression Scale (GDS-15)**, a validated screening tool for detecting depression in older adults [14]. GDS-15 scores ≥ 5 were considered indicative of clinically relevant depressive symptoms.

**Nutritional status**: Nutritional status was assessed using the **Mini Nutritional Assessment-Short Form (MNA-SF)** [15]. The MNA-SF categorizes patients as:

- Normal nutritional status,
- At risk of malnutrition,
- Malnourished.

### 2.6. Physical Reserve Assessment

Additional physical reserve parameters including calf circumference, gait speed, handgrip strength, and Tinetti Performance-Oriented Mobility Assessment were recorded as indicators of physical vulnerability and mobility performance [16].

**Tinetti performance test**: Balance and gait performance were evaluated using the **Tinetti Performance-Oriented Mobility Assessment**, which provides a composite score reflecting fall risk and mobility performance.

### 2.7. Statistical Analysis

All statistical analyses were performed using Jamovi (version 2.6) and the R statistical environment. Continuous variables were expressed as mean ± standard deviation **or** median (interquartile range) depending on distribution, and categorical variables were presented as frequencies and percentages. Normality was assessed using the Shapiro–Wilk test. Group comparisons were performed using Student’s *t*-test or Mann–Whitney U test as appropriate. Associations between continuous variables were evaluated using Spearman correlation analysis. To identify independent factors associated with polypharmacy, multivariable logistic regression analysis was performed. Variables included in the model were selected based on clinical relevance and prior literature and included age, sex, HbA1c, CGA parameters, physical reserve indicators, and frailty status. To reduce the risk of model overfitting, the number of variables included in the multivariable model was limited relative to the number of outcome events. Collinearity was assessed using variance inflation factors (VIFs). A two-sided *p*-value < 0.05 was considered statistically significant. To explore whether frailty modifies the association between glycemic control and polypharmacy, an interaction term between HbA1c and frailty status (HbA1c × frailty) was incorporated into the multivariable logistic regression model. A *p*-value < 0.05 for the interaction term was considered statistically significant.

### 2.8. Ethical Considerations

The study protocol was approved by the İnönü University Clinical Research Ethics Committee (approval number: 2021/2883). The study was conducted in accordance with the Declaration of Helsinki. Due to the retrospective design, the requirement for written informed consent was waived.

## 3. Results

A total of 278 older adults with T2DM were included (mean age 71.7 ± 6.0 years; 63.3% female). Polypharmacy (≥5 medications) was present in 54.7% of patients.

Patients with polypharmacy had significantly higher HbA1c and fasting glucose levels compared to those without polypharmacy (both *p* < 0.001). Poor glycemic control (HbA1c > 7.5%) was also more frequent in the polypharmacy group (*p* < 0.001). Hypertension was more prevalent among patients with polypharmacy, whereas BMI and dyslipidemia did not differ between groups (Table 1).

In the comprehensive geriatric assessment, patients with polypharmacy had lower ADL scores and slower gait speed (*p* = 0.007 and *p* = 0.036, respectively), while no significant differences were observed in IADL, cognitive function (SMMSE), depressive symptoms (GDS-15), nutritional status (MNA-SF), handgrip strength, calf circumference, or Tinetti score (Table 2).

In univariate analysis, higher HbA1c was strongly associated with polypharmacy (OR = 4.34, *p* < 0.001), whereas increasing age and higher ADL scores were associated with lower odds (Table 3). In the multivariable model, higher HbA1c levels remained independently associated with polypharmacy (OR = 4.99, 95% CI: 3.18–7.84, *p* < 0.001), whereas frailty status was not significantly associated with polypharmacy (OR = 0.58, *p* = 0.427) (Table 4).

Interaction analysis showed no significant HbA1c × frailty interaction (*p* > 0.05), indicating that the association between glycemic burden and polypharmacy was consistent regardless of frailty status.

**Figure 1:**
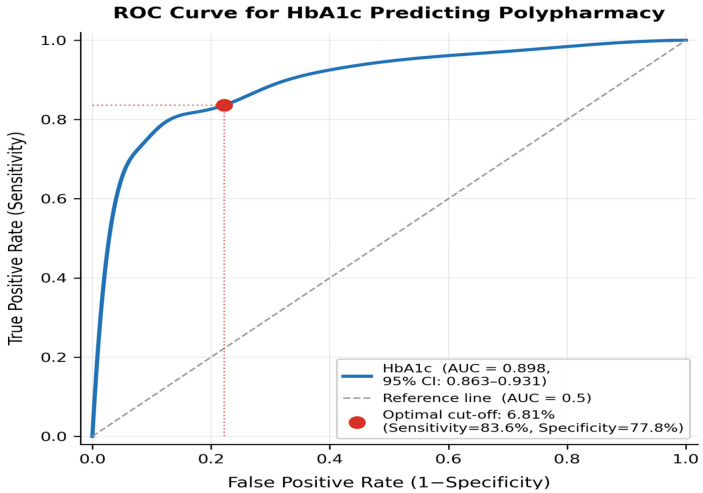
ROC curve of HbA1c for predicting polypharmacy in older adults with type 2 diabetes mellitus. HbA1c demonstrated strong discriminative performance (AUC = 0.898, 95% Cl: 0.863–0.931) with an optimal cut-off value of 6.81%, sensitivity 83.6%, and specificity 77.8%. Optimal cut-off values were determined using the Youden index.

ROC analysis demonstrated strong discriminative performance of HbA1c for predicting polypharmacy (AUC = 0.898, 95% CI: 0.863–0.931), with an optimal cut-off of 6.81%. Fasting glucose was analyzed separately and is reported in the text but was not included in Figure 1.

Frailty was present in 39.3% of patients but was not associated with medication count or polypharmacy prevalence (*p* = 0.345). HbA1c remained significantly associated with polypharmacy within both frail and non-frail subgroups.

## 4. Discussion

The principal finding of this study was that polypharmacy in older adults with T2DM appeared to be more strongly associated with markers of metabolic burden, particularly HbA1c levels, than with frailty status itself. Patients using polypharmacy had significantly higher HbA1c and fasting glucose levels, and HbA1c remained independently associated with polypharmacy in multivariate analysis. In contrast, frailty status was not independently associated with polypharmacy in this cohort, even though frailty and various physical performance parameters were assessed as part of a comprehensive geriatric evaluation. These findings suggest that in older adults with type 2 diabetes followed in tertiary geriatric clinics, drug burden may more closely reflect treatment intensity and metabolic dysregulation rather than frailty. While some geriatric parameters, such as walking speed and functional status, differed between groups in unadjusted analyses, these associations were less pronounced after multivariate adjustment.

The prevalence of polypharmacy in our cohort (54.7%) is consistent with figures reported in comparable populations of older adults with T2DM, where estimates have ranged from 50% to 78% depending on study setting and polypharmacy definition used [17,18]. The most striking finding of the present study was the magnitude of the association between glycemic dysregulation and polypharmacy: the mean HbA1c in the polypharmacy group was 8.5% versus 6.0% in the non-polypharmacy group, and each 1% increase in HbA1c was associated with a more than five-fold increase in the odds of polypharmacy in univariate analysis, while the fully adjusted multivariable model confirmed that HbA1c remained independently associated with polypharmacy. These findings suggest that in this cohort, polypharmacy appears to primarily reflect the progressive escalation of antidiabetic and cardiometabolic pharmacotherapy in response to poorly controlled hyperglycemia, rather than a generalized marker of geriatric vulnerability.

This interpretation aligns with the pathophysiology of T2DM as a progressively worsening metabolic condition requiring stepwise treatment intensification. Current guidelines recommend that when HbA1c targets are not met, the sequential addition of glucose-lowering agents is indicated, a process that inevitably increases medication count [19,20]. In older adults, this escalation is compounded by frequent co-prescription of antihypertensive agents, lipid-lowering drugs, and antiplatelet or anticoagulant therapies driven by cardiovascular comorbidities. In our study, hypertension was significantly more prevalent in the polypharmacy group, providing additional mechanistic support for this interpretation. The observation that 60.5% of patients in the polypharmacy group had poor glycemic control compared with only 4.0% in the non-polypharmacy group further underscores that treatment intensification in response to suboptimal metabolic control appears to be a major contributor to medication burden in this population.

From an endocrinological standpoint, the markedly elevated HbA1c levels observed in the polypharmacy group warrant careful interpretation in older adults with T2DM. Although HbA1c values exceeded the commonly recommended target range for functionally preserved older adults, individualized glycemic targets remain essential in geriatric diabetes care [21]. Importantly, hypoglycemia was observed exclusively in the polypharmacy group, highlighting the potential risks of intensive glucose-lowering regimens in this population. Hypoglycemia in older adults is associated with adverse outcomes including falls, fractures, cardiovascular events, and cognitive decline, particularly in patients with frailty or functional impairment [22].

From a clinical endocrinology perspective, these findings raise the question of whether treatment targets were appropriately individualized in this cohort. Current guidelines emphasize that HbA1c targets for older adults with T2DM should be stratified according to functional capacity, cognitive status, life expectancy, and comorbidity burden with less stringent targets (HbA1c 7.5–8.5%) acceptable for those with frailty, multimorbidity, or limited life expectancy [20]. The high prevalence of poor glycemic control alongside polypharmacy in our cohort may indicate that treatment was being intensified in pursuit of targets that were insufficiently individualized for a geriatric population. The simultaneous finding of 21.1% hypoglycemia in the polypharmacy group provides clinical evidence that treatment burden in some patients may have increased the risk of treatment-related adverse outcomes.

Frailty was identified in 39.3% of our cohort using the Fried phenotype, a prevalence broadly consistent with estimates in community-dwelling older diabetic populations reported in the literature, where the Fried phenotype identifies frailty in 25–45% of older adults with T2DM [23]. Notably, frailty in our cohort was characterized by markedly reduced handgrip strength, lower Tinetti balance and gait scores, greater depressive symptom burden, and worse nutritional status, with a striking predominance of female sex among frail patients. These patterns are consistent with the well-established phenotypic profile of frailty in older adults and highlight the multidimensional nature of the syndrome, which encompasses not only physical performance deficits but also psychonutritional vulnerability.

However, frailty did not independently predict polypharmacy in our cohort, nor was medication count significantly correlated with frailty status. Within both frailty strata, HbA1c remained the primary variable associated with polypharmacy (*p* < 0.001 in both subgroups), while physical performance and functional CGA measures did not differ significantly. These results stand in partial contrast with the prevailing literature, which has consistently described a significant and often bidirectional relationship between polypharmacy and frailty in broader older adult populations [24,25]. The discrepancy between our findings and previous reports may be related to the specific characteristics of older adults with T2DM, in whom treatment intensification is often driven by glycemic control requirements. In this context, the association between polypharmacy and frailty may become less pronounced, as medication burden appears to be more closely linked to metabolic dysregulation and age-related alterations in glucose metabolism than to frailty status alone [26]. This interpretation is supported by the observation that HbA1c levels remained significantly associated with polypharmacy across both frailty strata.

Although functional status and gait speed differed between groups in unadjusted analyses, these associations were no longer statistically significant after multivariable adjustment. This finding may reflect a bidirectional relationship: functional dependence may result partly from adverse drug events and anticholinergic burden associated with polypharmacy, while concurrently, the accumulated morbidity underpinning functional decline also drives prescription escalation. Notably, other core CGA domains including cognitive function (SMMSE), depressive symptoms (GDS-15), nutritional status (MNA-SF), and IADL did not differ significantly between polypharmacy groups. This pattern reinforces the interpretation that medication burden in this diabetic geriatric cohort is not a global expression of multidimensional frailty but rather a disease-specific therapeutic response.

No significant association was observed between polypharmacy and cognitive function in this cohort. Mean SMMSE scores were similar between groups, suggesting comparable levels of cognitive performance among patients with and without polypharmacy. Likewise, depressive symptom burden assessed by GDS-15 did not differ significantly between groups. These findings suggest that medication burden in older adults with T2DM may be more closely related to metabolic and treatment-related factors than to cognitive or affective status alone [22].

With respect to physical performance parameters, gait speed was significantly lower in the polypharmacy group (median 0.75 vs. 1.00 m/s, *p* = 0.036), and handgrip strength showed a borderline non-significant trend toward lower values (*p* = 0.100). These findings suggest a degree of physical vulnerability in patients with higher medication burden, though the direction of causality cannot be established in this cross-sectional design. Reduced gait speed below the critical threshold of 0.8 m/s which was present in the polypharmacy group is recognized as a marker of adverse outcome risk in geriatric populations and may reflect the combined impact of sarcopenic muscle dysfunction, polypharmacy-related sedation and orthostatic effects, and the functional consequences of poorly controlled T2DM itself, including peripheral neuropathy and cardiovascular deconditioning [7]. These parameters, while not independently driving polypharmacy in this cohort, represent important clinical targets for intervention and warrant systematic assessment in all older diabetic patients.

These findings may have implications for individualized medication management in older adults with T2DM. In this cohort, medication burden appeared to be more closely associated with poor metabolic control than with frailty status alone, suggesting that optimization of glycemic management may play an important role in reducing treatment burden. At the same time, medication-related decisions in older adults should remain individualized and guided by functional status, frailty, life expectancy, and patient preferences [27].

Frailty assessment remained clinically relevant in this cohort despite the absence of an independent association with polypharmacy. Frail patients demonstrated poorer physical performance, lower nutritional status, and higher depressive symptom burden, supporting the multidimensional nature of geriatric vulnerability [23]. These findings highlight the value of comprehensive geriatric assessment for individualized diabetes management in older adults, particularly when determining glycemic targets and balancing treatment burden against functional status and life expectancy. Current ADA Standards of Care recommend routine assessment of frailty, cognition, falls, depression, and polypharmacy in older adults with diabetes [23]. Interestingly, patients with polypharmacy were younger than those without polypharmacy. This finding may reflect differences in treatment intensity and prescribing patterns across age groups within the geriatric population.

This study has several strengths. Comprehensive geriatric assessment domains were evaluated together with objective physical performance measures including handgrip strength, gait speed, calf circumference, Tinetti score, and the Fried frailty phenotype. In addition, the integration of metabolic laboratory parameters with geriatric assessment data enabled simultaneous evaluation of metabolic and functional correlates of polypharmacy in older adults with T2DM.

The study also has important limitations that should be considered when interpreting the findings. The cross-sectional design precludes any causal inference about the direction of associations between polypharmacy, glycemic control, and frailty; longitudinal data would be required to determine whether polypharmacy contributes to frailty progression or whether frailty independently predicts subsequent medication escalation. The categorization of frailty into a binary frail versus non-frail dichotomy (combining pre-frail and robust into a single reference group) was dictated by the distribution of the available data, and this approach may have reduced the sensitivity of analyses to detect frailty-related gradients. The retrospective extraction of medication data from hospital records may not capture all over-the-counter or supplements use, potentially leading to underestimation of true polypharmacy prevalence. Individualized glycemic targets and clinician-specific treatment goals were not consistently available because of the retrospective study design. Importantly, the use of a uniform HbA1c threshold in this study should not be interpreted as recommending identical glycemic targets for frail and non-frail older adults. Rather, it was used as a standardized analytical definition of poor glycemic control, while individualized glycemic goals remain essential in geriatric diabetes care according to current guidelines [4,22]. Additionally, the study was conducted at a single tertiary referral center, which may limit the generalizability of findings to primary care or community settings where disease complexity and medication burden may differ. Finally, several key variables, including the specific antidiabetic agents prescribed, diabetes duration, renal function (eGFR), and comorbidity severity scores (e.g., Charlson Comorbidity Index), were not incorporated into the analysis, which represents a potential source of residual confounding.

## 5. Conclusions

In this cohort of older adults with T2DM, polypharmacy appeared to be more closely associated with markers of poor glycemic control, particularly HbA1c levels, than with frailty status itself. Although several geriatric assessment parameters differed in unadjusted analyses, frailty was not independently associated with polypharmacy after multivariable adjustment. These findings suggest that medication burden in older adults with T2DM may reflect treatment intensity and suboptimal glycemic control in addition to geriatric vulnerability.

## Figures and Tables

**Table 1:** Demographic and Clinical Characteristics by Polypharmacy Status.

Variable	Total (n = 278)	Polypharmacy (n = 152)	Non-Polypharmacy (n = 126)	*p*
Age (years), mean ± SD	71.7 ± 6.0	70.7 ± 5.6	72.8 ± 6.3	0.002
Gender, n (%)				0.188
Male	102 (36.7%)	50 (32.9%)	52 (41.3%)	
Female	176 (63.3%)	102 (67.1%)	74 (58.7%)	
Number of medications, mean ± SD	4.7 ± 2.1	6.1 ± 1.7	3.1 ± 0.9	<0.001
Hyperpolypharmacy (≥10 medications)	10 (3.6%)	10 (6.6%)	0 (0.0%)	—
HbA1c (%), mean ± SD	7.4 ± 1.9	8.5 ± 1.8	6.0 ± 1.0	<0.001
Fasting blood glucose (mg/dL), median (IQR)	—	234.5 (95.0)	102.5 (52.8)	<0.001
Poor glycemic control (HbA1c >7.5%), n (%)	96 (34.5%)	92 (60.5%)	5 (4.0%)	<0.001
Hypertension, n (%)	140 (50.4%)	91 (63.6%)	49 (39.5%)	<0.001
Dyslipidemia, n (%)	183 (65.8%)	106 (69.7%)	77 (61.1%)	0.106
Hypoglycemia, n (%)	32 (11.5%)	32 (21.1%)	0 (0.0%)	<0.001
BMI (kg/m^2^), mean ± SD	30.8 ± 7.5	31.0 ± 8.4	30.6 ± 6.6	0.930
Marital status				0.005
Married	191 (68.7%)	102 (67.1%)	89 (70.6%)	
Widowed/Other	87 (31.3%)	50 (32.9%)	37 (29.4%)	
Frailty (Fried), n (%)	108/275 (39.3%)	64/152 (42.1%)	44/123 (35.8%)	0.345

**Table 2:** Comprehensive Geriatric Assessment and Physical Performance Parameters by Polypharmacy Status.

CGA Domain	Total (n = 278)	Polypharmacy (n = 152)	Non-Polypharmacy (n = 126)	*p*
ADL score, median (IQR)	65.0 (25.0)	65.0 (28.8)	70.0 (20.0)	0.007
IADL score, median (IQR)	5.0 (2.8)	5.0 (2.8)	5.0 (2.8)	0.681
SMMSE score, mean ± SD	24.5 ± 5.2	24.2 ± 5.1	24.8 ± 5.4	0.113
GDS-15 score, median (IQR)	4.0 (7.0)	5.0 (6.8)	4.0 (7.0)	0.142
MNA-SF score, median (IQR)	12.0 (3.5)	12.0 (4.0)	12.0 (3.0)	0.171
Handgrip strength (kg), mean ± SD	24.8 ± 12.3	23.5 ± 11.1	26.5 ± 13.5	0.100
Calf circumference (cm), mean ± SD	41.1 ± 10.6	40.1 ± 8.0	42.4 ± 13.2	0.320
Gait speed (m/s), median (IQR) *	0.95 (0.70)	0.75 (0.70)	1.00 (0.70)	0.036
Tinetti score, median (IQR)	23.0 (6.0)	24.0 (6.8)	23.0 (7.5)	0.327
Frailty (Fried phenotype), n (%)	108/275 (39.3%)	64/152 (42.1%)	44/123 (35.8%)	0.345

GDS-15: Geriatric Depression Scale-15; MNA-SF: Mini Nutritional Assessment Short Form; SMMSE: Standardized Mini-Mental State Examination; IADL: Instrumental Activities of Daily Living; ADL: Activities of Daily Living. * Outlier values (>5 m/s) excluded. *p*-values from Mann-Whitney U test or chi-square test as appropriate.

**Table 3:** Univariate Binary Logistic Regression Analysis: Predictors of Polypharmac.

Variable	OR	95% CI	*p*
HbA1c	4.34	2.98–6.33	<0.001
Age	0.94	0.91–0.98	0.006
ADL	0.98	0.97–1.00	0.010
Gait speed	0.72	0.49–1.05	0.086
Handgrip	0.98	0.96–1.00	0.056
Calf circumference	0.98	0.96–1.00	0.099
MNA-SF	1.08	0.98–1.19	0.121
SMMSE	0.98	0.93–1.02	0.344
GDS-15	1.03	0.98–1.09	0.262
BMI	1.01	0.98–1.04	0.682
Frailty (frail vs. non-frail)	1.36	0.83–2.21	0.285

GDS-15: Geriatric Depression Scale-15; MNA-SF: Mini Nutritional Assessment Short Form; SMMSE: Standardized Mini-Mental State Examination; ADL: Activities of Daily Living. Outlier values (>5 m/s) excluded. *p*-values from Mann-Whitney U test or chi-square test as appropriate.

**Table 4:** Multivariable Logistic Regression for Polypharmacy (Adjusted for Frailty).

Variable	OR	95% CI	*p*
HbA1c	4.99	3.18–7.84	<0.001
Age	0.89	0.81–0.99	0.042
Female sex	0.98	0.45–2.14	0.956
Handgrip strength	0.99	0.95–1.02	0.373
Frailty (frail vs. non-frail)	0.583	0.154–2.211	0.427

## Data Availability

The data presented in this study are available on reasonable request from the corresponding author. The data are not publicly available due to ethical restrictions.
